# Mid-LAD Myocardial Bridging in a patient presenting with NSTEMI

**DOI:** 10.51894/001c.166027

**Published:** 2026-08-01

**Authors:** Muhammad Taimur

**Affiliations:** 1 Internal Medicine, Detroit Medical Center-Sinai Grace Hospital

## 127

### INTRODUCTION

Myocardial Bridging (MB) occurs when a portion of a coronary artery tunnels inside the myocardium. Left Anterior Descending (LAD) Artery is the most commonly implicated artery.

Typically, MB patients are asymptomatic on presentation but can be associated with acute coronary syndromes, exertional angina, cardiac arrhythmias, and sudden cardiac death. Most cases of MB are confirmed by autopsy with a prevalence of 86%. On coronary angiography (CA), the prevalence is lowered to 2-6%.

We present an atypical case of MB with NSTEMI diagnosed using CA in a 65-year-old man.

### CASE PRESENTATION

A 65-year-old male with a history of hypertension and left ventricular hypertrophy presented to the emergency department due to midsternal 7/10 crushing chest pain with mid-epigastric abdominal pain, nausea, nonbilious clear vomiting, and an episode of presyncope. Chest pain started abruptly after nausea and vomiting ensued four days before presentation. Chest discomfort worsened on exertion and relieved with rest without radiating to jaw or left arm.

The cardiac exam was benign. EKG showed a normal sinus rhythm with nonspecific ST-T wave changes. The initial troponin I high sensitivity was 129. CA was performed which demonstrated mid-LAD myocardial bridging leading to 80% obstruction due to systolic narrowing indicative of milking phenomenon. 2D echocardiogram showed an LVEF of 70%. Thus, the diagnosis of mid-LAD MB with systolic obstruction was identified as the cause of the patient’s symptoms. We started him on Aspirin 81mg, Amlodipine 10mg, and Atorvastatin 40mg. Chest pain subsided and we discharged him on hospital stay day 4 with outpatient follow-up with the cardiologist.

### DISCUSSION

Myocardial Bridging is generally a benign finding on CA and is not a cause for concern typically. Most patients with MB are asymptomatic. However, multiple cases have demonstrated stable or unstable angina, vasospastic angina, or acute coronary syndrome (ACS) due to MB complications. In our case, the patient presented with atypical symptoms, leading to an initial evaluation for acute mild gastritis. A negative workup resulted in an evaluation for a cardiac cause for his symptoms.

In conclusion, MB should be included as a differential when evaluating patients for ischemia with atypical symptoms.

